# Short-term results of a novel management of supracondylar fracture with coexisting osteoarthritis with bifold fixation and total knee arthroplasty

**DOI:** 10.1186/s42836-021-00098-0

**Published:** 2021-12-04

**Authors:** Nicholas A. Antao, Sanjay Londhe, Rajan Toor, Rajesh Shirishkar, Siddharth Aiyer

**Affiliations:** 1 Department of Orthopaedics Holy Spirit Hospital, Jeevan Data Hostel, Mahakali Caves Road, Andheri (E), Mumbai, Maharashtra 400093 India; 2 Indian Orthopaedic Research Group, Thane, Maharashtra 400604 India

**Keywords:** Total knee Arthroplasty, Supracondylar femoral fractures, Retrograde intramedullary solid locking nail (SIGN), Locking plate

## Abstract

**Purpose:**

Presence of supracondylar and periarticular femoral fracture with associated arthritis of knee poses a challenging situation to the orthopaedic surgeon. The results of fixation of fracture in osteoporosis are not very satisfactory and have complications. With fixation alone, they still cannot bear weight on affected leg due to severe disability of osteoarthritis. To make patient walk, conventionally three surgeries in the form of fracture fixation, removal of implant and total knee arthroplasty (TKA) needs to be done in staged manner. We propose a novel management in form of bifold fixation and simultaneous TKA.

**Methods:**

Eight cases (6 females, 2 males) of supracondylar femoral fractures with severe osteoarthritis of the knee and osteoporosis were primarily fixed with bifold fixation using SIGN nail (www.signfracturecare.org) and locking plate together with simultaneous total knee arthroplasty. There were five cases (2 males and 3 females) of grade 4 (Kellgren-Lawrence grading) osteoarthritis (OA) and three cases (all females) of severe rheumatoid arthritis (RA).

**Results:**

The mean age was 68 years and average time for full weight bearing was 6 days. Radiographic evidence of fracture union was achieved in 16.25 weeks. The mean Knee Society Score (KSS) and Western Ontario and McMaster Universities Osteoarthritis Index (WOMAC) score at 26 months was 83.13 and 22.13 respectively.

**Conclusions:**

Single stage combined bifold osteosynthesis with interlocking nail and locking plate together with total knee arthroplasty helps in one time management of these difficult injuries. It is a cost-effective and economically sound option and gives excellent results with good patient satisfaction.

## Introduction

Distal femoral fractures in the elderly are a major cause of morbidity and mortality [1, 2]. The reported incidence of distal femoral fractures is 6%. However, they have 1-year mortality rates ranging between 15 and 40%, similar to that of peri-trochanteric fractures [3–5]. These elderly patients often have concomitant secondary osteoarthritis of the knee joint, which impacts the eventual clinical and functional outcome [6, 7]. The coexistent osteoporosis may lead to considerable comminution at the fracture site with intra-articular, metaphyseal or diaphyseal extension [8].

Traditionally, distal femoral fractures have been treated with osteosynthesis [9, 10]. There has been a considerable evolution with time in the implants used for fixation. Blade plate and dynamic condylar screw with plate designs have given way more recently, and minimal invasive locking compression plates and retrograde distal femoral nails have become the preferred option for fixation [9–13]. Although these fixation techniques and devices give satisfactory results in younger individuals (less than 65 years), in the elderly (more than 65 years), achieving a stable fixation is challenging due to osteoporotic bone [7, 8, 14]. Even with a stable fixation, early weight bearing is not possible and partial weight bearing restrictions are difficult to execute for the elderly [7, 8]. Often presence of other comorbidities, poor cognitive function, movement disorders or neurological deficit can make early mobilization practically impossible [6, 8]. Prolonged recumbency in elderly patients has risk of further respiratory complications, pressure sores and eventually increase in morbidity and mortality [6, 15].

Primary arthroplasty has been increasingly used as a definitive management option for elbow, shoulder and hip trauma [16, 17]. The proponents of this management strategy cited benefits including immediate stability, return of joint movement and early restoration of ambulatory function [6, 7, 18].

The preliminary reports of the primary arthroplasty for the distal femoral fractures were made by Wolfgang *et al* and Bell *et al* in patients with rheumatoid arthritis and pre-existing osteoarthritis of the knee with a distal femoral fracture respectively [19, 20]. Since then, many authors have reported small series of primary arthroplasty for proximal tibia/distal femoral fractures in the elderly with promising results [7, 21]. The advantages quoted by these reports included early return to weight bearing and this strategy avoids a tedious reconstruction in the presence of a poor bone stock [6, 7, 18, 22]. However, often these series have used special prosthesis, including constrained designs, long stemmed femoral/tibial components and even modular tumour prosthesis [6, 8, 15, 21, 22].

The authors reported a short-term outcomes of a unique surgical strategy for the treatment of distal femoral fractures in elderly patients with coexistent arthritis. This strategy combines the concept of a stable fixation achieved with a distal femoral locking compression plate over a distal femoral interlocking intramedullary nail. This fixation is then supplemented by replacement of the articulating surfaces with a cruciate-sacrificing TKA prosthesis.

## Material and methods

A retrospective review of medical records was performed for eight patients undergoing distal femur fracture stabilization with the unique surgical strategy combining fracture fixation with replacement of the articular surfaces. The study was approved by the institutional review board and all ethical standards *as per* the Helsinki declaration and later amendments. Informed consent was obtained from all patients.

**Fig. 1:**
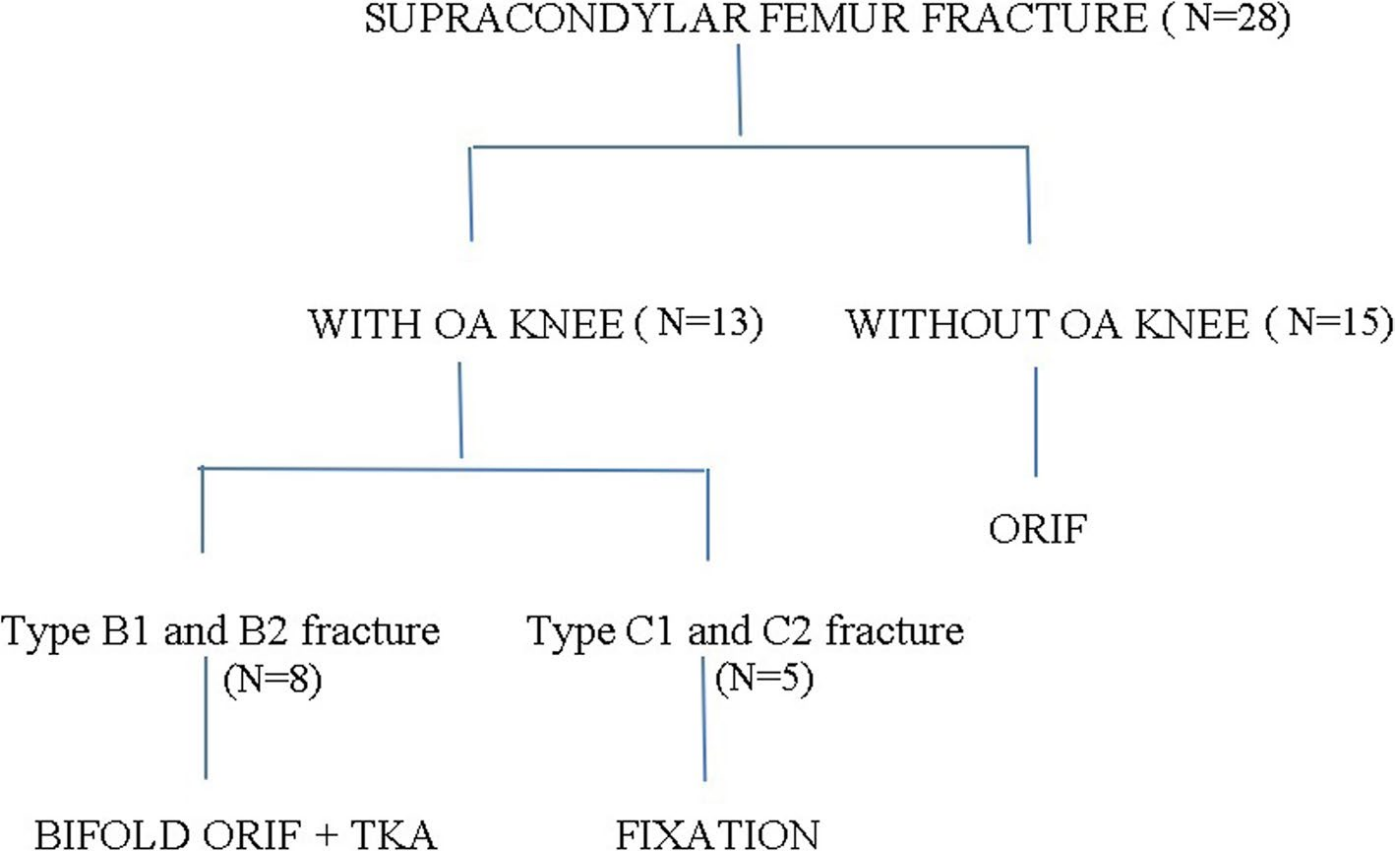
Flow chart of inclusion and exclusion criteria

Inclusion criteria included was patients with B1 and B2 type supracondylar fracture with coexistent OA. Patients with B1 & B2 type supracondylar fracture without coexistent OA and patients with C1 & C2 type supracondylar fracture with coexistent OA were excluded from the study (Fig. 1).

There were six women and two men with a mean age of 68 years (64-75 years). All eight patients had a distal femur fracture with associated arthritis in the knee, including five patients with primary osteoarthritis and three patients with rheumatoid arthritis. Two patients were socially-dependent and needed assistive devices for ambulation prior to fall. They were predominantly household ambulators. Six patients were independent community ambulators without the need of assistive devices.

### Implant and prosthesis design

All fractures were stabilized using a retrograde solid intramedullary locking SIGN nail (Richland, Washington, USA). Locking bolts were placed both proximally and distally when the distal fracture segment length was more than three times the diameter of the femur at the fracture site. If the distal fracture segment was smaller than the diameter of the fracture, no locking bolts were inserted into the distal femoral segment. Locking bolts were inserted into the proximal femoral segment in patients with osteoporosis and comminution to prevent collapse due to instability. A locking compression plate was inserted in all patients over the less comminuted surface, to offer better metaphyseal fracture segment stabilization. The TKA prosthesis used in all patients was a cruciate-sacrificing Zimmer Biomet AGC knee.

### Procedure

The surgical procedure was performed with the use of a well-padded tourniquet. A midline approach was used to expose the joint and the fracture site. The hematoma at the fracture site was evacuated and antibiotic wash (vancomycin with normal saline) was used during the procedure.

**Fig. 2:**
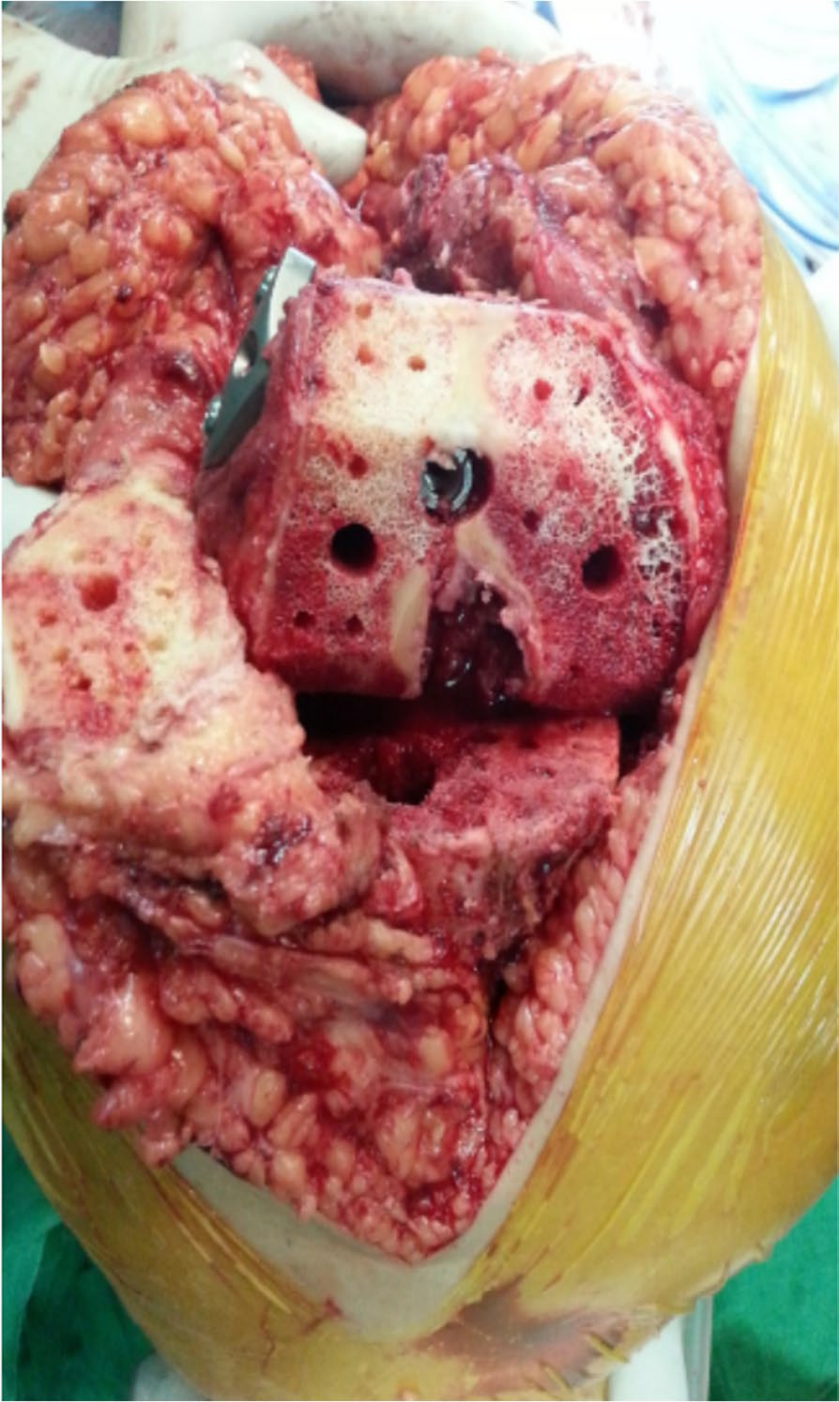
The bone cuts with RIMN (SIGN nail) and locking plate

The steps of the surgical technique were as follows: (1) Temporary fracture reduction with reduction clamps which was confirmed on C-arm; (2) The prerequisite tibial, femoral and patellar cuts were taken and trial reduction was performed; (3) Trial implants were removed and retrograde intramedullary nailing was performed (Fig. 2) by using a solid SIGN nail of appropriate diameter (8 or 9 mm). The nail of right length was selected so that the nail engages in the isthmus region of the femur; (4) A locking compression plate was applied to fix the metaphyseal region of the fracture with maximum alignment and stability; (5) TKA was performed by using a standard cruciate-sacrificing prosthesis without any constraint in the design.

### Postoperative protocol

In the immediate postoperative period, patients were maintained in a long leg knee brace. They were put on physiotherapy as early as postoperative day one. Isometric exercise of the quadriceps and hamstrings, along with continuous passive mobilization of the knee joint was initiated. The range of motion was gradually increased based on pain tolerance to achieve a 90° knee flexion by 2 to 4 weeks postoperatively as shown in Fig. 5. Patients were encouraged to do partial weight-bearing *as per* their pain tolerance for a period of 4 to 10 days, followed by full weight bearing.

### Assessment of outcomes

The clinical outcomes were assessed in terms of the range of motion achieved, time taken for weight bearing and length of hospital stay. The functional outcomes were assessed based on the Knee Society Scores (KSS) and the Western Ontario and Mc Master University Arthritis Score (WOMAC). WOMAC is a self-administered patient-reported outcome measure (PROM) consisting of 3 subscales, namely, pain (5 items), stiffness (2 items) and physical function (17 items). The test questions are scored on a scale of 0-4, 0 being none,1 mild, 2 moderate, 3 severe and 4 extreme. Hence, the range of the score is from 0 to 96. A higher WOMAC score indicates poor knee joint function and a lower score indicates good knee joint function. Radiographs were assessed for features of radiological union, coronal and sagittal alignment of the femoral and tibial components of the TKA. Survivorship of the TKA and any features of component loosening were noted. Mortality in the early perioperative period and 2-year mortality were recorded. Records were assessed for any complications occurring in the first 90-days follow-up and on subsequent follow-up for prosthetic joint infection and need of revision surgery.

## Results

In our series of eight patients (Table 1), the mean duration of follow-up was 26 months (24-32 months). The mean length of stay in the hospital was 10 days and mean time to full weight-bearing mobilization was 6 days (range, 4-10 days). There was no mortality in the eight patients in the 2-year follow-up.

**Table 1:** The preoperative, intraoperative and postoperative characteristics

Case no.	Age (years)	Sex	Type	Nail diameter (mm)	Weight bearing (days)	KSS	WOMAC	ROM (degrees)	Union time (weeks)
1	65	Female	OA	9	4	90	16	110	16
2	64	Female	OA	9	10	80	24	100	17
3	65	Female	OA	8	6	90	15	110	16
4	70	Male	RA	8	4	65	42	90	16
5	75	Female	RA	9	8	85	20	100	16
6	72	Female	OA	9	6	90	14	105	16
7	68	Female	RA	9	5	75	32	95	16
8	65	Male	OA	8	7	90	14	100	17

Data including fracture classification, type of anaesthesia, surgical time, blood loss and units of blood transfusion are shown in Table 2.

**Table 2:** Patient data of our series

Case no.	Fracture classification (AO)	Type of anaesthesia	Surgery time	Blood loss	Blood transfusion (Units of packed cell volume)
1	Type B1	Spinal+epidural	2.30 h	500 ml	1 PCV
2	Type B2	Spinal+epidural	2.35 h	450 ml	Nil
3	Type B2	Spinal+epidural	2.45 h	500 ml	1 PCV
4	Type B1	Spinal+epidural	2.30 h	300 ml	Nil
5	Type B2	Spinal+epidural	2.30 h	400 ml	Nil
6	Type B2	Spinal+epidural	2.50 h	300 ml	Nil
7	Type B1	Spinal+epidural	2.45 h	350 ml	Nil
8	Type B1	Spinal+epidural	2.40 h	400 ml	Nil

Six patients who were community ambulators without the need of assistive devices were able to return to pre-injury status. Two patients who were dependent household ambulators due to severe arthritis in opposite knee returned to pre-injury status.

**Fig. 3:**
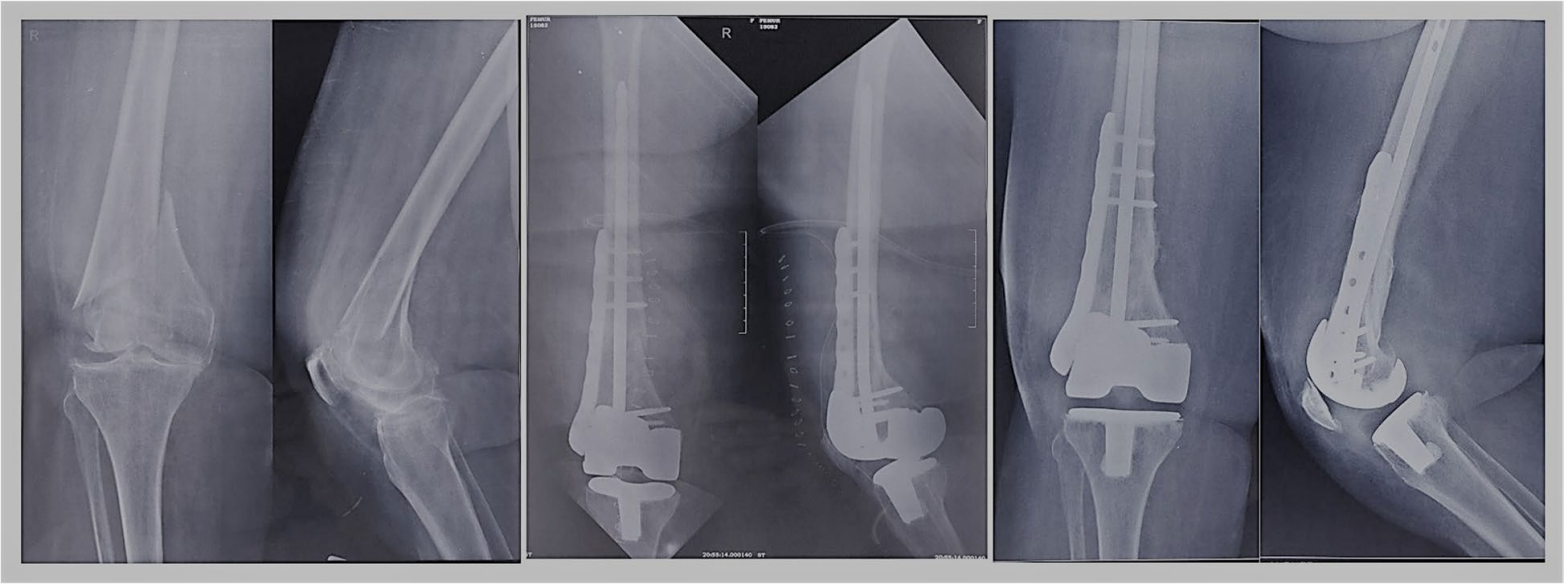
**a** Preoperative distal femural fracture with OA knee. **b** Postoperative fracture fixation with TKA, nail and locking plate. **c** The healed fracture with implant *in situ*

**Fig. 4:**
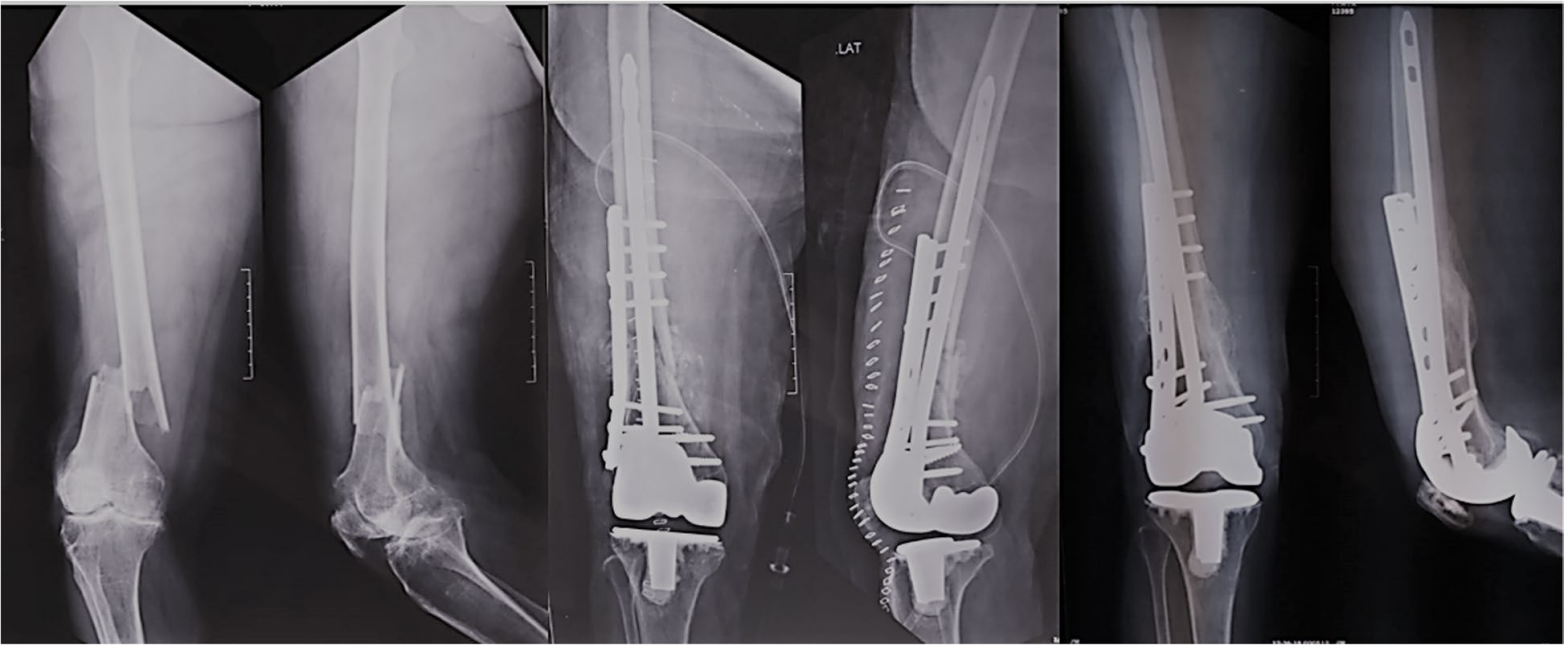
**a** Preoperative distal femural fracture with RA knee. **b** Postoperative fracture fixation with TKA, nNail and locking plate. **c** Healed fracture with implants* in situ*

**Fig. 5:**
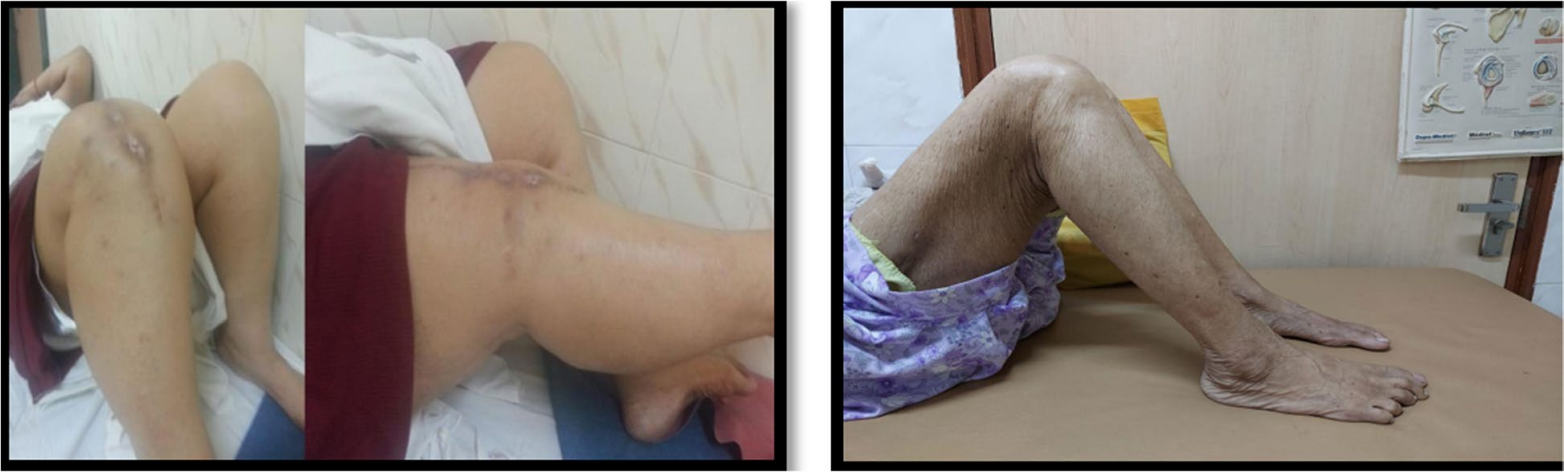
**a** Full final function of a patient with distal femural fracture with OA knee. **b** Full final function of the patient with distal femural fracture with RA knee

Radiological evidence of fracture union was achieved in all patients in a mean duration of 16.25 ± 0.46 weeks (16-17 weeks). No radiological evidence of loosening of prosthesis was noted at final follow-up (Figs. 2 & 3). The coronal and sagittal alignment of the femoral and tibial components were satisfactory in all patients. The clinical outcomes (Fig. 4) showed a mean range of knee flexion of 101.25° (90-110°). The mean KSS score was 83.13 ± 9.23 (65-90) and mean WOMAC score was 22.13 ± 10.15 (14-42). No complications such as surgical site infection, wound dehiscence, thromboembolic disease and prosthetic joint infection were noted Fig. 5.

## Discussion

Chen *et al* noted in a systematic review that Type A fracture (extra-articular fracture) can be treated with long-stemmed femoral components with limited internal fixation to augment the stability [21]. Type B and type C fracture,  which have intra-articular fracture extension, often need treatment with modular prosthesis or constrained design prosthesis based on the integrity of the collateral ligament attachments and extent of metaphyseal comminution [23]. The present series offers a unique approach where an unconstrained total arthroplasty prosthesis can be used provided a supplementary internal fixation is performed with an intramedullary solid nail and a locking compression plate at the same time.

Modular and constrained prostheses have a higher risk of aseptic loosening, mechanical failure and a lower survivorship compared to unconstrained TKA design in degenerative osteoarthritis [6, 8]. The life expectancy of the patient must be taken into consideration when offering the constrained prosthesis/modular mega prosthesis to patients with distal femoral fractures and arthritis [8]. Parratte *et al* presented the only multicentre retrospective series analysing 26 patients operated in eight centres where unconstrained prosthesis was used in 9 patients, 12 patients needed medullary stem extenders and 5 patients had constrained hinge prostheses [24].

Mean range of knee flexion achieved in the present series was 101.25° (90-110°), which was comparable to the range achieved in other retrospective series by Nau *et al*, Wui *et al* and Boureau *et al* [18, 25, 26]. The mean KSS was 83.13 in our series and was comparable to the findings by Malviya *et al*, who reported a mean KSS of 90 in 26 elderly patients treated with primary arthroplasty for periarticular fractures [14]. Wui and co-workers documented a mean KSS of 87 in a series of 10 patients treated with arthroplasty following knee fractures at a 2-year follow-up [26].

Distal femoral fractures in the elderly have been associated with an increased 1-year mortality and in some series, have shown a rate comparable to that of hip fractures [6, 8, 21]. In our series, no mortality was noted in the first 2-year follow-up. However, Appleton and others reported one of the largest series of 52 patients of distal femoral fractures undergoing joint replacement procedures with a documented 1-year mortality of 42% [6]. The mean age of their cohort study was considerably higher (82 years) than the mean age of 68 years in our series.

One of the advantages of primary arthroplasty for distal femoral fractures is early return to weight bearing and ambulatory function [22, 23]. This is important for the elderly in whom prolonged recumbency is associated with thromboembolic events, pressure sores and respiratory complications [23]. Mean time to ambulation in our series was 6.25 days and passive knee range of movements were started within the first 48 h. This early return to ambulatory function and no postoperative thromboembolic events were an encouraging outcome in the present series.

Reports by Appleton and Rosen have both documented early return to pre-injury ambulatory status after TKA in distal femoral fractures with arthritis [6, 22]. However, Boureau *et al* have reported a decline in the level of autonomy among the operated patients when compared with the pre-injury ambulatory function [18]. In our series, all the patients returned to pre-injury ambulatory status, including the two patients who needed walking aids even before injury.

In patients with intra-articular distal femoral fractures, a suboptimal reduction can lead to late post-traumatic arthritis [7, 23]. Outcomes of knee arthroplasty in the post-traumatic scenario are inferior to primary arthroplasty for degenerative arthritis [6, 27]. An additional procedure of implant removal is often necessary prior to embarking upon arthroplasty and surgical procedure may need to be staged with the use of constrained design prothesis, long-stemmed femoral component or hinged revision prostheses [23].

Our study has certain limitations. The first limitation is that the average age of the patients in our series was younger than that reported by Boureau *et al* and Paratte *et al*. Hence, we are regularly following up these patients to see the long-term results of this novel technique. Another limitation of our study is that we did not compare this technique with the traditional method of management for these fractures, namely, open reduction & internal fixation, then implant removal and the final third surgery in the form of TKA. We propose to do a formal prospective study comparing these two methods. Since we avoided two further surgeries, it reduced the total cost to the patient. The third limitation is that we used WOMAC score as the patient-reported outcome measure (PROM). This is in variance to the already described studies in which different outcome measures (PROM) were used.

One of the possible downsides of this technique is the theoretically increased risk of infection due to extra surgical time. However, we did not encounter infection in our patients.

These limitations are in line with most published literature on primary arthroplasty for distal femoral fractures [7, 21, 23]. We hereby presented the short-term outcomes of a unique strategy which, to the best of our knowledge, has not been previously reported.

## Conclusion

In summary, the present series documented encouraging results with the use of a combination of surface replacement and bifold internal fixation for the treatment of distal femoral fractures with arthritis. These functional results were comparable with previously published literature on the use of primary arthroplasty for the treatment of distal femoral fractures. Using a bifold internal fixation has allowed for use of a standard TKA prosthesis without the need of any advanced prosthetic designs.

## Data Availability

Transparent. Funding: This research did not receive any specific grant from funding agencies in the public, commercial, or not-for-profit sectors.

## Abbreviations

TKA: Total Knee Arthroplasty
KL: Kellgren Lawrence
OA: Osteoarthritis
RA: Rheumatoid Arthritis
KSS: Knee Society Score
WOMAC: Western Ontario and McMaster Universities Osteoarthritis Index
RIMN: Rimmed Intramedullary Nail
